# Comparison of Dietary Intakes of 7-Year-Old Children Enrolled in Observational Birth Cohort Studies on the Isle of Man and in South-west England

**DOI:** 10.3390/nu9070724

**Published:** 2017-07-08

**Authors:** Ellen M. Tweney, Pauline M. Emmett, Jean Golding, Stephanie Goodfellow, Caroline M. Taylor

**Affiliations:** 1 Centre for Child and Adolescent Health, School of Social and Community Medicine, Oakfield House, Oakfield Grove, University of Bristol, Bristol BS8 2BN, UK; et15141@bristol.ac.uk (E.M.T.); p.m.emmett@bristol.ac.uk (P.M.E.); jean.golding@bristol.ac.uk (J.G.); stephgoodfellow@gmail.com (S.G.); 2 Castletown, Isle of Man

**Keywords:** Isle of Man, ALSPAC, dietary food record, children, ELSPAC, diet, free sugars

## Abstract

There is concern regarding the amount of fruit and vegetables consumed and high sugar intakes in children’s diets. Regional dietary differences in the British Isles could underlie variations in health outcomes, but little is known about these differences. Our aim was to compare diets of children enrolled in observational birth cohort studies in the Isle of Man (IoM-ELSPAC) and in south-west England (ALSPAC). Dietary intakes were assessed by 3-day food records in IoM and ALSPAC at an age of 7 years. Comparisons of mean daily nutrient, and food and food group intakes were made between the studies and with UK national dietary guidelines. Diets in both regions were adequate for most nutrients except dietary fibre, but in both groups intake of free sugars was three times higher than the UK recommended maximum. There were differences between the two regions, particularly higher energy, protein, and carbohydrate intakes in IoM. IoM children consumed greater amounts of red meat, bread, full-fat milk, and sugar-sweetened drinks. IoM children had higher intakes of energy and some nutrients and food groups than ALSPAC children, and similar low intakes of fruits and vegetables. Children’s diets in both regions could be improved, particularly considering the increasing prevalence of childhood obesity and the UK recommendation to lower the intake of free sugars.

## 1. Introduction

Much is known about the diets of children across the UK from several studies, including the National Diet and Nutrition Survey (NDNS) [1], and in Ireland from the National Children’s Food Survey (NCFS) [2]. There are very few data, however, on regional differences in overall diets and food consumption. The NDNS does provide data on regional differences in consumption of individual food items but the geographical areas covered are very large. In depth comparisons between more closely defined geographical areas may provide important insights into regional differences in diet and health.

The Isle of Man (IoM) is an understudied area of the British Isles and there may be important differences in diet compared with the mainland. The IoM is a self-governing Crown dependency situated in the Irish Sea with a population of approximately 76,000 in 2001, comprising about 50% native Manx (Celtic origin) with the remainder mostly UK and Irish immigrants [3]. It is generally a prosperous community [4]; however, there are inequalities, reflected in the proportions of households in fuel poverty (>10% of gross income spent on fuel) (17%) compared with England (10%) [5], and health inequalities in particular are similar to those found in areas of north-west England (Goodfellow (1995), Whitehead (1990) and Isle of Man Chief Registrar, cited in Goodfellow and Northstone [6]). Comparisons between the diets consumed in the IoM and other parts of the British Isles could be informative in developing regional public health advice.

The present study takes advantage of data from two birth cohort studies with a similar design that were initiated at the same time: the IoM Study and the Avon Longitudinal Study of Parents and Children (ALSPAC) in south-west England. Both studies included assessment of children’s diets at the age of 7 years using similar 3-day dietary records [7], thus providing an opportunity to investigate regional differences between the cohorts. It is possible that children’s diets differ between these regions because IoM children have been shown to have higher levels of obesity than children in ALSPAC at 7 years of age, especially boys [6]. There are also difference in maternal diets during pregnancy: those in the IoM have been shown to be higher in fat and lower in fresh vegetables, and IoM mothers were shorter and heavier than those in ALSPAC [6].

The primary aim of this study therefore was to document the food and nutrient intakes of 7-year-old children in the IoM and to compare them with those in ALSPAC to assess regional differences in diet. The secondary aim was to compare the nutrient intakes of the children in the IoM and ALSPAC with the UK dietary reference values (DRV) using the recommended and lower reference nutrient intakes (RNI and LRNI) [8,9]. This information could be of value in developing healthcare policies and practice in the IoM.

## 2. Methods

### 2.1. Birth Cohort Studies: Isle of Man and ALSPAC

**Figure 1:**
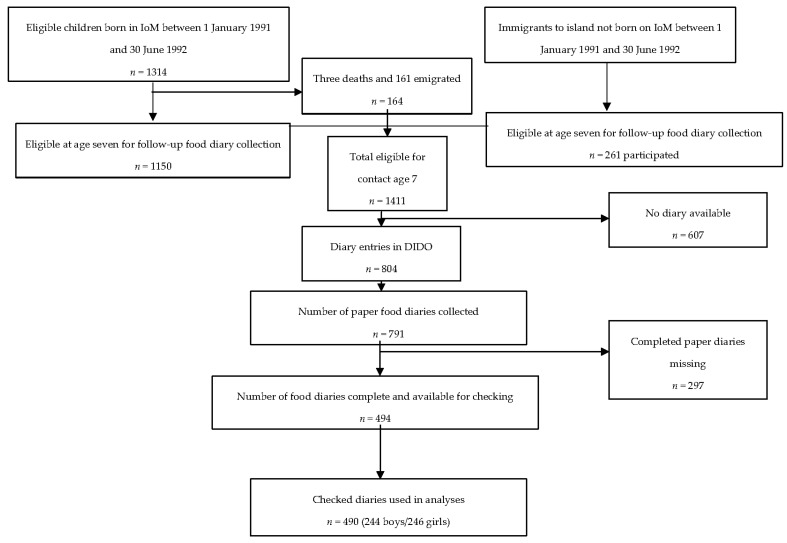
Flowchart showing how final study numbers for data analysis were obtained.

The Isle of Man birth cohort study formed part of the European Longitudinal Study of Pregnancy and Childhood (ELSPAC) aiming to collect data from eight European countries during pregnancy and to follow the offspring of the pregnancy through childhood. The aim of the ELSPAC was to investigate environmental and genetic influences on the health, behaviour, and development of children, and to identify factors that might improve child health. The IoM was invited to join as it represents a distinct and self-contained population where all resident pregnant women could potentially be included. All pregnant women resident on the island with an expected date of delivery in the 18-month period of January 1991–June 1992 were invited to enrol. The eligible cohort was formed from 1314 live births. At the age of 7 years there was an additional phase of recruitment to cover all children who had subsequently moved to the IoM (Figure 1). Full details of the data collection in IoM and a study flowchart can be accessed from the cohort profile [10] and at the study website [11].

ALSPAC is the UK arm of ELSPAC. A total of 14,541 pregnant women resident in Avon with expected dates of delivery from April 1991 to December 1992 were recruited. Similarly to the IoM study, a second phase of recruitment at the age of 7 years (Phase II) generated an additional 452 participants; the phases of enrolment are described in more detail in the cohort profile [12]. The study website contains details of all the data that are available through a fully-searchable data dictionary [13].

### 2.2. Demographic Data Collection

Questionnaires completed by mothers and/or their partners during the pregnancy and after the birth of the study child were identical in the IoM and ALSPAC cohorts. Data collected included maternal age, parity (defined as the number of previous pregnancies with a live birth or stillbirth), ethnicity, pre-pregnancy BMI, and marital status.

Copies of the questionnaires used in ALSPAC can be accessed and downloaded [14].

### 2.3. Dietary Data Collection: IoM and ALSPAC

The collection of dietary data at the age of 7 years was via parental completion of a 3-day food record carried out prior to the visit to the corresponding clinic. Parents/caregivers were asked to record all the food and drink their children consumed over two weekdays and one weekend day describing the amounts in household measures [7]. In the IoM, the majority of parents had the food records checked by staff during the clinic visit and extra details about the foods were added to the records as necessary. This degree of scrutiny by staff was not possible in ALSPAC, but parents completed a short questionnaire that covered the details most likely to be missing from the food records.

### 2.4. Data Processing: IoM and ALSPAC

The paper records were coded by a fieldworker using a specialist program DIDO (Diet In, Diet Out) that is designed for the direct entry of dietary records and uses a hierarchical menu of food codes and portion sizes [15]. The DIDO coding method has the advantage of greater accuracy, speed, consistency, and efficient data handling, and affords greater data accessibility for checking, than manual systems [15]. The data were then checked against the original records by a different fieldworker and any errors corrected.

For the IoM 494 paper copies of the food records out of 791 were available to check against DIDO entries. After appropriate corrections, comparisons were made with the uncorrected records showing that the correction process made an appreciable difference to nutrient intakes and amounts of particular foods consumed (data not shown). Therefore, only the records where checking had been possible were used in the present analyses. Of the 494 records, 65 were for children newly recruited at the age of 7 years and intakes from these food records were compared with those from the original cohort (Supplementary Tables S1 and S2). Only very minor differences were found so all records were included in the analysis. Clinic data were missing in four cases and so these food records were excluded, leaving 490 (see Figure 1). For ALSPAC all data entries were checked against the original food records.

The nutrient database used for the analyses was based on the fifth edition of McCance and Widdowson’s food tables [16] with supplements. The nutrient contents of foods not in the original database were obtained from the NDNS nutrient databank or calculated from manufacturers’ information. The method for calculating free sugars (non-milk extrinsic sugars (NMES)) was taken from the NMES definition provided by the Department of Health [8]. The data were used to calculate mean daily nutrient intakes for each child as described by Emmett, et al. [17]. The foods and food groups included in the analyses were selected based on those used in NDNS [1]. Misreporting of energy intake was identified based on estimated energy requirements predicted from age, sex, and weight measurements in the 7-year clinic, with allowance for growth and a standard level of physical activity; a reported energy intake of between 79% and 121% of estimated energy requirements was defined as being a plausible report [18,19,20].

To check the adequacy of the children’s diets, nutrient comparisons were made with UK DRVs for 7-year-olds, where available [8,9], and the UK Scientific Advisory Committee on Nutrition [21] recommendation of 20 g AOAC fibre per day (equivalent to 15 g non-starch polysaccharide (NSP) fibre per day) for 5–11-year-olds.

### 2.5. Statistical Analyses

Data analysis used SPSS version 23 (IBM Corp., Armonk, NY, USA). To account for multiple comparisons, multivariate analysis of variance (MANOVA) was used to evaluate whether nutrient or food group intakes differed between the IoM and ALSPAC. If the MANOVA was statistically significant, then comparisons between the two cohorts by sex were conducted using analysis of variance (ANOVA) for mean nutrient and energy-adjusted macronutrient intakes and for food groups by weight and contribution to energy. Comparisons with DRVs were made by calculating the percentage of participants in each group falling below the threshold level. To check that the findings were not biased by misreporting status, comparisons of nutrient and food group intakes were repeated using only the plausible reporters in both cohorts.

## 3. Results

Data from the IoM were based on 490 food records (244 boys and 246 girls) and from ALSPAC based on 7087 food records (3593 boys and 3494 girls). The demographic characteristics of both cohorts are shown in Table 1. There were no demographic data available from children enrolled into the IoM study at the age of 7 years (*n* = 65). The mothers of IoM children with checked food diary data were slightly older and more likely to be married than those without (Table 1). The demographic characteristics for IoM compared with ALSPAC for participants with food record data at the age of 7 years were broadly similar except that the IoM mothers were slightly younger than the ALSPAC mothers. IoM children had a greater mean gestation length than ALSPAC children.

There were no differences in nutrient intakes between children recruited at birth and those recruited at 7 years in the IoM (Supplementary Table S1). Similarly, there were no differences in food item intakes with the exception of total spreads (Supplementary Table S2). In IoM under-reporting was identified in 15.6% of children and over-reporting in 15.4% (Supplementary Table S3); equivalent levels in ALSPAC were 12.4% and 13.0%, respectively. Absolute nutrient intakes were different between the three misreporting groups, as expected; however, among the energy-adjusted macronutrients, the percentage of energy from carbohydrates and free sugars were not different between the groups, for either sex.

Using all the available checked food records, mean energy intake was 3.5% higher in boys and 5.5% higher in girls in IoM than ALSPAC (Table 2). This was accounted for by higher mean percentage of energy from protein and carbohydrate and absolute intakes of both protein and carbohydrate in IoM than ALSPAC. The mean percentage of energy from fat among IoM children, both girls and boys, was lower than in ALSPAC but the amount of fat consumed was similar. The percentage of energy from free sugars and absolute intake of free sugars was different only among the girls, with a higher mean intake in IoM. Results from comparisons between plausible reporters only confirmed these findings for energy, protein, and carbohydrates; however, higher mean intakes of free sugars were found in the IoM for both boys and girls (Table 2; Supplementary Table S4). Due to the high correlation of most micronutrients with energy intake, mean intakes of micronutrients in IoM tended to be higher than those in ALSPAC. Retinol was the exception to this with a lower mean intake in boys in IoM.

Compared with UK recommendations [9], the estimated average requirement (EAR) for energy was exceeded by more than half the children in both cohorts. In general, more IoM children exceeded the RNIs for the nutrients [8], although the differences between the two cohorts were not great (Table 3). Almost all children in both cohorts failed to reach the UK recommendation for fibre [21]. Consumption of wholemeal bread, wholegrain cereals, vegetables, and fruit was low. Twice as many ALSPAC children as IoM children were below the RNI for niacin (Table 4). Double the proportion of ALSPAC children were below the LRNI for zinc compared with IoM children.

Considerably more bread was eaten by children in IoM than ALSPAC (26% and 34% more in boys and girls, respectively; Table 4). This was mostly white bread in both locations. The volume of full-fat milk consumed was greater in the IoM in both sexes than in ALSPAC. In ALSPAC children were as likely to consume semi-skimmed milk as full-fat milk, whereas IoM children were more likely to consume full-fat milk. Almost all children from both locations ate meat, but there was a significant difference in the total amount and type of meat eaten (Table 4). About twice as much beef was eaten by children in the IoM than ALSPAC; most other meats, but not processed meats, were also eaten in greater amounts in IoM. Amounts of fruits and vegetables consumed were no different between the cohorts; however, potatoes (other than fried, roast or chips) were consumed by a greater proportion of children in IoM. There were major differences in soft drink consumption between the cohorts. A great proportion of children consumed fruit juice in the IoM than in ALSPAC, similarly with sugar-sweetened soft drinks consumption. In contrast, ALSPAC children were more likely to drink diet/low-energy soft drinks, but to offset that were more likely to consume sugar in confectionery. Similar differences in food group intakes were found when comparing plausible reporters between the cohorts (Supplementary Table S5). From reviewing the completed food records, it appeared that traditional meals such as meat with vegetables and potatoes and roast dinners were common in IoM children’s diets, and milk was frequently consumed at bedtime, often with cereal.

There were important differences in the amount of energy derived from the various food groups (Table 5). The milk, bread, and meat groups each contributed 9%–12% of energy with greater contribution from each group in IoM than ALSPAC. Sweet foods contributed about one-fifth of the total energy in IoM, whereas their contribution was nearer one-quarter in ALSPAC. Each of the four types of sweet foods provided 4%–5% of the energy. Four percent of IoM children’s energy intake was from high-sugar drinks (double that of ALSPAC children), and fruit juice also contributed more energy in the IoM.

## 4. Discussion

We found important differences between the two regional cohorts in energy and macronutrient consumption: IoM children had significantly higher mean energy intakes, specifically from greater mean protein and carbohydrate intakes in both sexes. Higher intakes of meat, full-fat milk and bread in the IoM were major contributors to this. There was very little evidence that children in either region had inadequate intakes of any nutrients apart from a lack of dietary fibre and an excess of free sugars. It was striking that soft drink consumption was very different between the cohorts with IoM children drinking greater amounts of sugar-sweetened varieties, and also fruit juices. The reasons for differences in food intakes between the regions have not been studied, but may be driven by disparities in culture, food availability and pricing, and health education levels.

As with many other similar studies of childhood diet in Europe [22,23] and the USA [24], and including ALSPAC [7], there are concerns about the types of foods consumed, particularly the low amounts of fruits and vegetables and high amounts of sweet foods, such as confectionery, biscuits, and sugar-sweetened soft drinks [25,26]. In both IoM and ALSPAC, children’s fruit and vegetable intakes were equivalent to about half of the five-a-day portions recommended [27]. There were very few children in either region whose free sugars intake was below the previous UK recommended maximum of 10% of daily energy intake, suggesting that achieving the revised lower target of 5% [21] will prove to be extremely challenging. Furthermore, about one-quarter of the total energy intake of the children was from sugar-sweetened foods and drinks (Table 5).

Some similarities can be seen between the diets of children in the IoM and those of children in post-war 1950s Britain [28] where, in both groups, “traditional” meals (roast meat, potatoes, and vegetables) were commonly eaten as a main meal at home. In addition, bread and milk formed two of the main components of the diets of both sets of children. Types of meat consumed in IoM children also shows some parallels with diets from the 1950s. Although intakes were generally lower in the early 1950s due to continued rationing, red meat made up a greater proportion of total meat consumption than for children in the 1990s from the NDNS [28]. This was mirrored in the differences found in the proportion of beef and lamb eaten between the IoM and ALSPAC. Of the children who ate meat in the ALSPAC cohort, the greatest consumption was of chicken products.

IoM and ALSPAC are in very different regions of the British Isles: the IoM lies in the Irish Sea between north-west England, Scotland, and Ireland, while ALSPAC is based in the Bristol area of south-west England. IoM comprises towns with a large rural area, whilst the area represented in ALSPAC comprises a large city (Bristol) with associated suburbs and a rural fringe. Thus, the dietary differences may reflect regional and environmental factors. Although the NDNS has investigated regional difference in children’s diets and found only marginal variations in nutrient intakes and types of foods eaten [1], the large diverse geographical areas assessed and the fact that Ireland is not included make it difficult to form meaningful comparisons with IoM. Data from the Irish NCFS [2], in which the diets and eating habits of 5–12-year-old children in 2003–2004 were analysed, show some similarities with those of IoM children. The differences in food intakes that were most marked between the IoM and ALSPAC are also evident as differences between the Irish NCFS and ALSPAC. In both IoM and Ireland, children ate more red meat, in particular beef, than ALSPAC children; similarly, full-fat milk consumption was much higher, as was the overall amount of potatoes eaten. Soft drink intake was more difficult to compare as the categorisation was not equivalent; however, intake of diet drinks compared with regular carbonated drinks was lower in Ireland, which was similar to the IoM, but in contrast to ALSPAC. This suggests that overall the diets of IoM children tended to be more like those of Irish children than of children in south-west England.

The differences between the pregnant women’s diets in the IoM compared with ALSPAC (pregnant women in the IoM consumed more fat and less fresh vegetables than those in ALSPAC [6]) were not seen in their children.

### 4.1 Strengths and Limitations

A key strength of the IoM study is that it took place in a self-contained island population and the survey design included all pregnant women, enabling the greatest demographic spread possible. The two main communities of the native Manx and the “incomers” were represented (see footnote to Table 1). Previous research has found the island to be a good location for studies, generating reliable results and well suited to epidemiological research [10]; however, the size of the cohort is relatively small compared with other similar studies, including ALSPAC. Dietary analysis for the IoM was further constrained by the availability of only a proportion of paper records for checking against the coded food records; therefore, the number of food records for the IoM (*n* = 490) was considerably smaller than for ALSPAC (*n* = 7087) and was slightly less representative of the population.

The use of estimated food records has been found to be an effective and reasonably accurate method of measuring an individual’s food consumption [29]. For dietary assessment in children, 3-day food records with parents as proxy reporters are the most accurate method of estimating total energy intake in 4–11-year-olds [30]. Using dietary reference values to assess the nutritional adequacy of children’s diets is limited by the paucity of evidence on which the values are based. As with any food records, however, there is a possibility of misreporting of intake, whether intentionally or accidentally. Under-reporting may be the result of omission of items, particularly if the child ate foods when not with the parent. Over-reporting may occur if the parent provided food but the child did not consume it all without the parent’s knowledge. Most the food records in IoM were checked by a field worker at the follow up clinic to try to overcome this problem. In Supplementary Tables S4 and S5 we have presented comparison data between IoM and ALSPAC in plausible reporters only that substantiate our findings using the full cohorts. With regard to the generalisability of results in ALSPAC, the NDNS has a rolling programme of assessing diet in children that has been used as a comparator: dietary intakes in ALSPAC 7-year-olds were very similar to those of 7–10-year-old children in 1997 in the NDNS [7]. In addition, an update and extension of the NDNS carried out between 2008 and 2012 in children aged 1.5–18 years has been used to assess whether diet in children at different ages has changed since the time of the ALSPAC data collection: the intake of fruit was slightly higher in 2008–2012, although intake of free sugars was very similar to that in ALSPAC children [31].

Finally, the issue of multiple comparisons for dietary variables was addressed by testing the data with MANOVA before univariate ANOVA tests. To fully adjust for multiple testing, a more stringent alpha would be set at <0.001. Under these conditions data with *p*-values <0.05 but ≥0.001 would not be regarded as being significant.

## 5. Conclusions

Children in both regions were consuming nutritionally-adequate diets in general, except that dietary fibre intake was low due, in part, to a lack of fruit and vegetables; in addition, sugar-containing foods and drinks were regularly consumed and contributed to a high energy intake. Differences in food intakes between the regions may be driven by disparities in culture, food availability and pricing, and health education levels. There is a need for education for parents, children, schools, and health professionals on how to achieve a healthy balanced diet. There is evidence that where children’s diets lack fruits, vegetables, and wholegrain foods, and regularly include confectionery, cakes, biscuits, white bread, and sugary drinks, there is an increased risk of obesity [32]. Furthermore, the high intake of free sugars among these children highlights the challenge posed by the recent UK recommendation to reduce the intake of free sugars to below 5% of energy [21].

## Figures and Tables

**Table 1:** Comparison of demographic characteristics between children in IoM with and without food records at 7 years of age, and children with food records in ALSPAC.

Characteristic	IoM ^a^			ALSPAC	*P* for IoM Included vs. ALSPAC Included
	With a Checked Food Record (*n* = 490)	Without a Checked Food Record (*n* = 921)	*P* value for IoM Included vs. IoM Excluded	With a Checked Food Record (*n* = 7087)	
Sex, *n* (%)					
Male	244 (49.8%)	466 (52.5%)	0.340	3593 (50.7%)	0.699
Female	246 (50.2%)	422 (47.5%)		3494 (49.3%)	
Birth weight (g)	3453 ± 525 (*n* = 421)	3410 ± 559 (*n* = 886)	0.179	3445.6 ± 533.7 (*n* = 6633)	0.438
Gestation length (weeks)	39.8 ± 1.7 (*n* = 421)	39.9 ± 1.7 (*n* = 886)	0.386	39.5 ± 1.8 (*n* = 6710)	<0.001
Maternal characteristics					
Parity			0.228		
0	188 (50.4%)	288 (46.5%)		2985 (46.4%)	0.131
≥1	185 (49.6%)	332 (53.5%)		3450 (53.6%)	
Marital status					
Never married	58 (15.2%)	166 (26.1%)	<0.001	848 (12.9%)	0.317
Widowed/Divorced/Separated	20 (5.2%)	41 (6.4%)		295 (4.0%)	
Married	304 (79.6%)	430 (67.5%)		5432 (8.3%)	
Age (years)					
<20	20 (5.2%)	53 (8.2%)	0.007	487 (6.9%)	0.013
20–24	71 (18.4%)	170 (26.4%)		893 (12.6%)	
25–29	145 (37.6%)	213 (33%)		2648 (37.4%)	
30–34	108 (28.0%)	148 (22.9%)		2218 (31.3%)	
≥35	42 (10.9%)	61 (9.5%)		841 (11.9%)	
BMI (kg/m^2^)	23.1 ± 3.7 (*n* = 367)	23.0 ± 3.77 (*n* = 611)	0.792	22.9 ± 3.7 (*n* = 6094)	0.364
Ethnicity ^b^					
White	373 (96.9%)	625 (97.2%)	0.770	6371 (98.3%)	0.035
Other	12 (3.1%)	18 (2.8%)		108 (1.7%)	

^a^ IoM demographic data from children enrolled at age 7 years not available (*n* = 65 for those with checked food records). For IoM: Manx 188 (50.4%), English 138 (37.0%), Irish 20 (5.4%), Scots 15 (4.0%), Other 12 (3.2%). ANOVA or chi square test.

**Table 2:** Energy and nutrient intakes from 3-day food records in 7-year-old children: comparison between IoM and ALSPAC by sex.

	IoM		ALSPAC	
	Boys	Girls	Boys	Girls
*n*	244	246	3593	3494
Energy (MJ)	7.70 (7.53, 7.88) *	7.26 (7.09, 7.43)^†††^	7.44 (7.39, 7.48)	6.88 (6.84, 6.92)
% from protein	14.0 (13.7, 14.3) ***	13.6 (13.3, 13.9) ^†^	13.3 (13.2, 13.4)	13.3 (13.2, 13.4)
% from fat	33.9 (33.3, 34.4)	34.3 (33.8, 34.9)	35.3 (35.2, 35.5) ***	35.6 (35.5, 35.7) ^†††^
% from saturated fat	14.1 (13.7, 14.4)	14.0 (13.6, 14.3)	14.2 (14.1, 14.3)	14.3 (14.2, 14.3)
% from carbohydrate	55.0 (54.3, 55.6) ***	54.9 (54.3, 55.6)^†††^	51.4 (51.3, 51.6)	51.2 (51.0, 51.3)
% from free sugars	18.2 (17.4, 19.0)	19.0 (18.4, 19.8)^†††^	17.5 (17.3, 17.7)	17.4 (17.2, 17.6)
Protein (g)	64 (62, 66) ***	59 (57, 58) ^†††^	58 (57, 58)	53 (53, 54)
Fat (g)	70 (67, 72)	66 (64, 69)	71 (71, 72)	66 (66, 67)
Carbohydrate (g)	253 (246, 259) ***	238 (232, 244) ^†††^	239 (237, 240)	220 (218, 221)
Free sugars (g)	84 (80, 88)	83 (79, 87) ^†††^	82 (81, 83)	75 (74, 76)
Fibre (g NSP)	11.1 (10.6, 11.5)	10.2 (9.8, 10.5)	10.8 (10.7, 10.9)	10.0 (9.9, 10.1)
Retinol equivalents (µg)	615 (578, 652)	603 (570, 636)	687 (673, 700) **	670 (651, 689)
Thiamin (mg)	1.5 (1.4, 1.5)	1.3 (1.2, 1.4)	1.5 (1.5, 1.66)	1.4 (1.35, 1.41)
Riboflavin (mg)	1.8 (1.7, 1.8)	1.5 (1.4, 1.5)	1.7 (1.73, 1.77)	1.5 (1.53, 1.57)
Niacin equivalents (mg)	31 (30, 32) ***	28 (27, 29) ^†††^	27 (27.2, 27.6)	25 (24.9, 25.3)
Vitamin B_6_ (mg)	2.0 (2.0, 2.1) ***	1.9 (1.8, 1.9) ^†††^	1.8 (1.81, 1.86)	1.7 (1.67, 1.70)
Vitamin B_12_ (µg)	4.1 (3.8, 4.3)	3.7 (3.5, 3.9)	3.9 (3.82, 3.94)	3.6 (3.50, 3.64)
Folate (µg)	209 (201, 218)	190 (184, 197)	205 (203, 207)	190 (188, 192)
Vitamin C (mg)	92.0 (83.1, 100.3) **	98.0 (90.3, 105.7) ^†††^	80.6 (78.7, 82.5)	80.0 (78.2, 81.9)
Vitamin D (µg)	2.4 (2.2, 2.6)	2.3 (2.2, 2.4)	2.5 (2.4, 2.5)	2.3 (2.26, 2.33)
Calcium (mg)	891(855, 927) ***	787 (757, 818) ^†^	824 (814, 833)	751 (742, 760)
Iron (mg)	9.3 (9.0, 9.6) ***	8.3 (8.1, 8.6) ^††^	8.7 (8.7, 8.8)	7.9 (7.9, 8.0)
Zinc (mg)	7.1 (6.9, 7.4) ***	6.2 (6.0, 6.4) ^†††^	6.4 (6.3, 6.4)	5.8 (5.7, 5.8)
Selenium (µg)	64 (62, 67) ***	60 (57, 62) ^†††^	55 (54, 55)	50 (49, 50)
Iodine (µg)	158 (149, 168)	136 (129, 142)	156 (153, 158)	139 (137, 141)

Values are shown per day. NSP, non-starch polysaccharide. MANOVA results (Wilks’ lambda *p* < 0.001 for boys and *p* < 0.001 for girls) justified post hoc ANOVA. Statistically significant difference by sex between IoM and ALSPAC (ANOVA): boys: * *p* ≤ 0.05, ** *p* ≤ 0.01, *** *p* ≤ 0.001; girls: ^†^ *p* ≤ 0.05; ^††^ *p* ≤ 0.01; ^†††^ *p* ≤ 0.001.

**Table 3:** Comparisons of nutrient intakes for children in the IoM and ALSPAC assessed by food records at 7 years of age with UK dietary reference values for children aged 7–10 years.

	UK Reference Intake Age 7–10 Years ^a^		% Children Below RNI		% Children Below LRNI	
	RNI	LRNI	IoM	ALSPAC	IoM	ALSPAC
Energy (kJ)						
Boys	6900 ^b^	-	28.3	35.7	-	-
Girls	6400 ^b^	-	23.2	36.7	-	-
Fibre (g NSP)	15.3 ^c^	-	92.5	92.3	-	-
Retinol equivalents (µg)	500	250	38.1	34.9	4.5	4.4
Thiamin (mg/4.2 MJ)	0.4	0.2	0.4	0.8	0.0	0.1
Riboflavin (mg)	1.0	0.5	8.9	11.9	0.2	0.6
Niacin equivalents (mg/4.2 MJ)	6.6	4.4	8.1	16.9	0.2	1.5
Vitamin B_6_ (µg/g protein)	15	11	0.0	0.5	0.0	0.1
Vitamin B_12_ (µg)	1.0	0.6	0.6	1.7	0.0	0.5
Folate (mg)	150	75	21.1	23.7	0.4	0.7
Vitamin C (mg)	30	8	11.1	16.2	0.0	0.5
Calcium (mg)	550	325	11.5	19.5	0.8	1.6
Iron (mg)	8.7	4.7	52.0	62.1	1.0	2.0
Zinc (mg)	7	4	63.0	74.4	4.3	8.3
Selenium (mg)	30	16	2.6	6.9	0.4	0.4
Iodine (mg)	110	55	30.0	34.1	1.8	2.9

RNI, reference nutrient intake, intakes above this amount will almost certainly be adequate; LRNI, lower reference nutrient intake, intakes below this amount will almost certainly be inadequate. NSP, non-starch polysaccharide. IoM boys *n* = 244, girls *n* = 246; ALSPAC boys *n* =3593, girls *n* = 3494. ^a^ Values are shown per day; Department of Health [8] unless indicated otherwise. ^b^ Estimated average requirement (EAR) at age 7 years [9]. ^c^ Scientific Advisory Committee on Nutrition [21].

**Table 4:** Comparisons of daily food group intakes from food records in 7-year-old children in the IoM and ALSPAC by sex.

Food Groups	Mean Weight (g per Day) and Consumers (%)			
	IoM		ALSPAC	
	Boys *n* = 244	Girls *n* = 246	Boys *n* = 3593	Girls *n* = 3494
**Total bread**	93 (98) ***	91 (98) ^†††^	74 (97)	68 (97)
White bread	78 (95) ***	76 (94) ^†††^	59 (90)	54 (89)
Brown bread	5 (10) **	3 (11)	2 (8)	2 (9)
Wholemeal bread	7 (14)	4 (12 )	9 (20)	7 (20)
High-fibre white bread	2 (6)	3 (8)	1 (3)	1 (2)
Other bread	1 (5) *	4 (13)	3 (11)	3 (12)
**Total cereals**	35 (95)	23 (86)	35 (90)	28 (85) ^††^
High-fibre breakfast cereal	17 (62)	10 (48)	18 (55)	14 (48) ^†^
Other breakfast cereal	17 (70)	13 (61)	17 (61)	14 (59)
Biscuits	20 (84)	19 (81)	22 (83)	19 (82)
Cakes, buns, fruit pies	21 (58)	23 (65)	29 (70) ***	28 (70) ^††^
Puddings, ice cream	40 (68)	42 (76)	45 (70)	44 (73)
**Total milk**	344 (97) ***	258 (94)	282 (96)	236 (94)
Full-fat milk	219 (70) ***	155 (66) ^†††^	145 (55)	114 (51)
Semi-skimmed milk	97 (41)	88 (42)	128 (54) **	113 (54) ^††^
Skimmed milk	20 (10) ***	10 (7) ^†^	5 (5)	5 (5)
Cheese	11 (57)	13 (65)	11 (54)	12 (58)
Yoghurt, fromage frais	39 (56)	34 (57)	38 (55)	35 (55)
Eggs/egg dishes	9 (29)	8 (27)	8 (26)	8 (29)
**Total spreads (includes butter)**	9 (87)	8 (89)	12 (91)	11 (91)
Butter	2 (23)	2 (23)	3 (21)	2 (23)
Full fat spreads	5 (56)	5 (62)	8 (71)	7 (70)
Low fat spreads	2 (21)	1 (19)	1 (9)	1 (10)
**Total meat**	106 (98) ***	98 (100) ^†††^	82 (96)	76 (95)
Pork	7 (17) *	5 (14)	5 (17)	5 (17)
Beef	21 (43) ***	19 (40) ^†††^	13 (31)	13 (31)
Chicken, turkey dishes	23 (58) ***	22 (55) ^†††^	17 (50)	16 (50)
Lamb	10 (17) ***	8 (16) ^††^	5 (16)	5 (14)
Bacon, ham	10 (55) *	10 (52) ^††^	8 (46)	7 (45)
Sausages	11 (43)	11 (44) ^††^	10 (40)	8 (35)
Burgers/ kebabs	4 (15)	2 (13)	4 (15)	3 (12)
Pies	7 (14)	6 (15)	5 (15)	5 (16)
Coated chicken, turkey	11 (38)	11 (38)	10 (30)	10 (32)
Other meat products	2 (12)	3 (13)	3 (15)	2 (14)
**Total fish**	12 (35)	14 (43)	14 (40)	15 (44)
Oily fish	3 (13)	5 (20)	3 (13)	4 (18)
Coated white fish	7 (22)	7 (24)	8 (27)	8 (26)
Other fish	2 (5)	3 (7)	2 (6)	2 (7)
**Total vegetables**	45 (83)	50 (87)	42 (87)	54 (89)
Salad/raw vegetables	5 (24)	6 (31)	6 (31)	8 (38)
Carrots, cooked	11 (53)	10 (52)	11 (52)	10 (51)
Green leafy vegetables	8 (36)	9 (36)	10 (42)	10 (45)
Peas	7 (43)	7 (44) ^†^	6 (35)	6 (35)
Green and runner beans	1 (9)	1 (9)	1 (10)	2 (12)
Tomatoes, tinned/cooked raw	3 (14)	6 (24)	5 (20)	6 (26)
Other cooked vegetables	9 (43)	8 (42)	9 (40)	9 (41)
Baked beans	19 (38)	16 (35)	19 (41)	15 (36)
Potatoes, fried, roast, chips	50 (85)	47 (81)	50 (82)	47 (81)
Other potatoes	41 (70) ***	36 (69) ^††^	30 (57)	30 (57)
**Total fruit**	79 (76)	82 (81)	79 (76)	83(82)
Citrus fruit	11 (21)	8 (22)	9 (19)	12 (24)
Apples and pears	33 (48)	35 (57)	31 (50)	30 (52)
Bananas	19 (39)	17 (37)	18 (33)	16 (32)
Other fresh fruit	15 (30)	19 (43)	18 (36)	22 (43)
Canned fruit	2 (5)	2 (7)	2 (6)	3 (8)
Savoury snacks, crisps	17 (79)	17 (83)	18 (82)	17 (83)
Chocolate, confectionery	13 (64)	13 (68)	17 (73) ***	15 (72)
Sugar, confectionery	5 (40)	6 (51)	11 (53) ***	12 (57) ^†††^
Sugar, preserves, sweet spreads	7 (68)	6 (67)	9 (70) **	8 (68) ^††^
Fruit juice	117 (61) *	135 (66) ^†††^	89 (49)	89 (51)
Soft drinks, sugar-sweetened	224 (81) ***	217 (87) ^†††^	129 (53)	112 (50)
Soft drinks, diet or low calorie	141 (59)	117 (54)	337 (81) ***	311 (82) ^†††^

MANOVA results (Wilks’ lambda *p* < 0.001 for boys and *p* < 0.001 for girls) justified post hoc ANOVA. Statistically significant difference by sex between IoM and ALSPAC (ANOVA): boys: * *p* ≤ 0.05, ** *p* ≤ 0.01, *** *p* ≤ 0.001; girls: ^†^ *p* ≤ 0.05; ^††^ *p* ≤ 0.01; ^†††^ *p* ≤ 0.001.

**Table 5:** Percentage of energy derived from individual food items from food records in 7-year-old children: comparison of IoM with ALSPAC by sex.

Foods	IoM		ALSPAC	
	Boys *n* = 244	Girls *n* = 246	Boys *n* = 3593	Girls *n* = 3494
Milk	11.2 (10.3, 12.0) ***	9.1 (8.3, 9.9) ^†††^	9.2 (9.0, 9.4)	8.2 (8.0, 8.5)
Bread	12.4 (11.5, 13.2) ***	12.8 (12.1, 13.6) ^†††^	10.1 (9.9, 10.3)	10.0 (9.8, 10.2)
Total meat	12.3 (11.5, 13.0) ***	11.9 (11.1, 12.6) ^†††^	10.2 (10.0, 10.4)	10.1 (9.9, 10.3)
Beef, pork, lamb, poultry	5.7 (5.1, 6.3) ***	5.4 (4.8, 6.0) ^†††^	4.1 (4.0, 4.2)	4.2 (4.1, 4.4)
Processed meat	6.5 (5.9, 7.2)	6.4 (5.8, 7.0)	6.1 (5.9, 6.3)	5.9 (5.7, 6.1)
Potatoes (all)	7.4 (6.8, 8.0)	7.4 (6.8, 8.0)	7.1 (7.0, 7.3)	7.4 (7.3, 7.6)
Fried, roast and chips	5.2 (4.6, 5.7)	5.3 (4.8, 6.0)	5.5 (5.3, 5.7)	5.6 (5.5, 5.8)
Other	2.3 (1.9, 2.6) ***	2.1 (1.8, 2.4) ^†^	1.6 (1.5, 1.7)	1.8 (1.7, 1.9)
Sweet foods (all)	18.5 (17.5, 19.5)	20.2 (19.1, 21.3)	24.0 (23.7, 24.3) ***	24.1 (23.8, 24.4) ^†††^
Confectionery	5.4 (4.8, 5.9)	5.9 (5.3, 6.4)	7.9 (7.7, 8.0) ***	7.8 (7.6, 8.0) ^†††^
Buns, cakes and pastries	4.3 (3.7, 4.9)	4.9 (4.3, 5.6)	5.9 (5.7, 6.1) ***	6.2 (6.0, 6.4) ^†^
Puddings including ice-cream	3.5 (3.0, 4.0)	4.1 (3.6, 4.6)	4.2 (4.1, 4.4) *	4.5 (4.3, 4.6)
Sweet biscuits	5.4 (4.8, 5.9)	5.2 (4.7, 5.8)	6.0 (5.8, 6.2)	5.6 (5.5, 5.8)
Fruit juice	2.5 (2.1, 2.9) *	3.1 (2.6, 3.5) ^†††^	1.9 (1.8, 2.0)	2.0 (1.9, 2.1)
Soft drinks with sugar	3.9 (3.4, 4.4) ***	4.0 (3.5, 4.5) ^†††^	2.3 (2.1, 2.4)	2.1 (2.0, 2.2)
Diet soft drinks	0.2 (0.1, 0.3)	0.3 (0.2, 0.4)	0.3 (0.2, 0.3)	0.3 (0.2, 0.3)

MANOVA results (*p* < 0.001 for boys and *p* < 0.001 for girls) justified post hoc ANOVA. Values are mean (95% CI). Statistically significant difference by sex between IoM and ALSPAC (ANOVA): boys: * *p* ≤ 0.05, ** *p* ≤ 0.01, *** *p* ≤ 0.001; girls: ^†^ *p* ≤ 0.05; ^††^ *p* ≤ 0.01; ^†††^ *p* ≤ 0.001.

## Supplementary Materials

The following are available online at www.mdpi.com/2072-6643/9/7/724/s1. Table S1: Comparison of nutrient intakes and percentage of energy derived from macronutrients for children in the IoM, from food records in 7-year-old children, by age of recruitment, Table S2: Comparison of mean weight (g/day) of individual food items consumed, from food records in 7-year-old children, by recruitment age of child in IoM, Table S3: Effect of under- and over-reporting on nutrient intakes in the IoM from food records in 7-year-old children, Table S4: Comparison of macronutrient intakes between plausible reporters in the IoM and ALSPAC from food records in 7-year-old children by sex, Table S5: Comparison of food group intakes (g/day) between plausible reporters in the IoM and ALSPAC from food records in 7-year-old children, by sex.
