# Supplementary material for: Comparison of Dietary Intakes of 7-Year-Old Children Enrolled in Observational Birth Cohort Studies on the Isle of Man and in South-west England
Source: Nutrients. 2017 Jul 8;9(7):724. doi: 10.3390/nu9070724 (PMC5537838; doi:10.3390/nu9070724)
Supplement: Supplementary file 1 [file nutrients-09-00724-s001.pdf]

Taylor et al. Comparison of dietary intakes of 7-year-old children enrolled in observational birth cohort studies on the Isle of Man and in south-west England

Supplementary Table S1 Comparison of nutrient intakes and percentage of energy derived from macronutrients for children in the IoM, from food records in 7-year-old children, by age of recruitment

	IoM	
	**Recruited at birth**	**Recruited age 7 years**
n	425	65
Energy (MJ)	7.48 (7.34, 7.61)	7.49 (7.19, 7.78)
% from protein	13.8 (13.6, 14.0)	13.7 (13.1, 14.3)
% from fat	34.1 (33.6, 34.5)	34.2 (33.1, 35.2)
% from saturated fat	14.0 (13.8, 14.3)	14.0 (13.6, 14.3)
% from carbohydrate	54.9 (54.4, 55.4)	55.0 (53.7, 56.3)
% from free sugars	18.8 (18.2, 19.3)	19.8 (18.2, 21.4)
Protein (g)	61 (60, 63)	61 (57, 64)
Fat (g)	68 (66, 70)	68 (64, 72)
Carbohydrate (g)	245 (240, 250)	246 (235, 257)
Free sugars (g)	83 (80, 86)	88 (80, 96)
Fibre (g NSP)	10.7 (10.4, 11.0)	10.4 (9.6, 11.1)
Retinol equivalents (µg)	613 (586, 640)	580 (522, 638)
Thiamin (mg)	1.4 (1.4, 1.5)	1.3 (1.2, 1.4)
Riboflavin (mg)	1.6 (1.6, 1.7)	1.6 (1.6, 1.8)
Niacin equivalents (mg)	30 (29, 30)	30 (28, 31)
Vitamin B_6 (mg)	1.9 (1.9, 2.0)	1.9 (1.8, 2.0)
Vitamin B_{12} (µg)	3.9 (3.7, 4.0)	3.8 (3.4, 4.2)
Folate (µg)	200 (194, 205)	202 (187, 216)
Vitamin C (mg)	94 (88, 100)	99 (83, 115)
Vitamin D (µg)	2.4 (2.2, 2.5)	2.2 (2.0, 2.4)
Calcium (mg)	835 (809, 861)	864 (799, 929)
Iron (mg)	8.8 (8.6, 9.1)	8.5 (8.0, 9.0)
Zinc (mg)	6.7 (6.5, 6.9)	6.4 (5.9, 6.8)
Selenium (µg)	62 (61, 64)	59 (55, 64)
Iodine (µg)	147 (140, 153)	148 (131, 165)

Values are mean (95% CI).
Values are shown per day.
NSP, non-starch polysaccharide.
Statistically significant difference between recruited at birth and at age 7 years (ANOVA): *p≤0.05, **p≤0.01, ***p≤0.001.

Supplementary Table S2 Comparison of mean weight (g/day) of individual food items consumed, from food records in 7-year-old children, by recruitment age of child in IoM

**Food groups**	**IoM**	
	**Recruited at birth**	**Recruited age 7 years**
n	425	65
**Total bread**	94*	80
White bread	78	69
Brown bread	4	5
Wholemeal bread	6	4
Other bread	3	3
**Total cereals**	29	29
High-fibre breakfast cereal	14	14
Other breakfast cereal	15	15
Biscuits	19	22
Cakes, buns, fruit pies	22	23
Puddings, ice cream	41	42
**Total milk**	301	300
Full-fat milk	191	160
Semi-skimmed milk	87	126
Skimmed milk	15	13
Cheese	11	14
Yoghurt, fromage frais	36	38
Eggs/egg dishes	9	8
**Total spreads (includes butter)**	8**	6
Butter	2	1
Full fat spreads	5	4
Low fat spreads	2	1
**Total meat**	103	92
Pork	6	8
Beef	21	16
Chicken, turkey dishes	22	22
Lamb	9	8
Bacon, ham	14	11
Sausages	11	10
Burgers/ kebabs	3	1
Pies	7	6
Coated chicken, turkey	11	12
Other meat products	2	2
**Total fish**	12	17
Oily fish	4	6
Coated white fish	7	7
Other fish	2	4
**Total vegetables**	48	45
Salad/raw vegetables	5	7
Carrots, cooked	10	8
Green leafy vegetables	9	8
Peas	7	5
Green and runner beans	1	2
Other cooked vegetables	9	8
Baked beans	18	13
Potatoes, fried, roast, chips	48	52
Other potatoes	39	33
**Total fruit**	79	89
Citrus fruit	9	11
Apples and pears	34	36
Bananas	18	19
Other fresh fruit	16	21
Canned fruit	2	1
Savoury snacks, crisps	17	16
Chocolate, confectionery	13	15
Sugar, confectionery	5	6
Sugar, preserves, sweet spreads	6	8
Fruit juice	123	145
Soft drinks, sugar sweetened	222	206
Soft drinks, diet or low calorie	130	119

Values are weight (g) per day.

Statistically significant difference between those recruited at birth and those recruited at age 7 (ANOVA): *p≤0.05, **p≤0.01

Supplementary Table S3 Effect of under- and over-reporting on nutrient intakes in the IoM from food records in 7-year-old children

	IoM all		IoM under-reporters		IoM plausible reporters		IoM over-reporters	
	Boys	Girls	Boys	Girls	Boys	Girls	Boys	Girls
n	244	246	45	29	161	166	33	40
Energy (MJ)	7.70 (7.53, 7.88)	7.26 (7.09, 7.43)	6.14 (5.80, 6.48)	5.55 (5.19, 5.91)	7.74 (7.59, 7.90)	7.21 (7.07, 7.35)	9.61 (9.31, 9.92)	8.82 (8.35, 9.29)
% from protein	14.0 (13.7, 14.3)	13.6 (13.3, 13.9)	15.2 (14.4, 16.0)	14.7 (13.7, 15.8)	13.6 (13.3, 13.9)	13.5 (13.2, 13.8)	13.9 (13.2, 14.5)	13.0 (12.4, 13.7)
% from fat	33.9 (33.3, 34.4)	34.3 (33.8, 34.9)	32.5 (31.1, 34.0)	33.3 (31.7, 34.9)	34.0 (33.3, 34.6)	34.0 (33.3, 34.6)	35.3 (33.9, 36.7)	36.5 (34.9, 38.1)
% from saturated fat	14.1 (13.7, 14.4)	14.0 (13.6, 14.3)	13.1 (12.3, 13.8)	13.3 (12.4, 14.3)	14.1 (13.7, 14.5)	13.9 (13.5, 14.3)	15.1 (14.5, 15.9)	14.7 (13.8, 15.7)
% from carbohydrate	55.0 (54.3, 55.6)	54.9 (54.3, 55.6)	55.1 (53.3, 56.8)	54.8 (52.8, 56.9)	55.3 (54.5, 56.0)	55.4 (54.6, 56.1)	53.7 (51.8, 55.6)	53.3 (51.5, 55.0)
% from free sugars	18.2 (17.4, 19.0)	19.0 (18.4, 19.8)	15.8 (14.1, 17.5)	16.7 (14.3, 19.1)	17.9 (17.0, 18.7)	18.5 (17.7, 19.3)	17.5 (15.2, 19.8)	18.7 (17.0, 20.4)
Protein (g)	64 (62, 66)	59 (57, 58)	55.6 (51.7, 59.5)	48.8 (44.3, 53.3)	63.1 (61.1, 65.1)	58.2 (56.6, 59.8)	79.5 (75.4, 83.6)	68.3 (64.2, 72.3)
Fat (g)	70 (67, 72)	66 (64, 69)	53.2 (49.1, 57.3)	49.7 (44.8, 54.5)	69.9 (67.9, 72.0)	65.1 (63.3, 66.9)	90.0 (85.3, 94.8)	85.5 (79.3, 91.7)
Carbohydrate (g)	253 (246, 259)	238 (232, 244)	201.9 (189.1, 214.7)	180.5 (170.0, 191.0)	255.5 (249.7, 261.3)	238.6 (232.9, 244.4)	308.6 (293.7, 323.5)	280.9 (263.1, 298.8)
Free sugars (g)	84 (80, 88)	83 (79, 87)	60.9 (52.9, 69.0)	57.1 (49.3, 64.9)	86.7 (82.2, 91.2)	83.6 (79.5, 87.7)	105.4 (91.3, 119.5)	103.3 (91.6, 115.1)
Fibre (g NSP)	11.1 (10.6, 11.5)	10.2 (9.8, 10.5)	10.0 (8.9, 11.0)	7.8 (6.9, 8.8)	11.0 (10.4, 11.6)	103.4 (10.0, 10.8)	13.0 (11.9, 14.1)	11.0 (10.0, 12.0)
Retinol equivalents (µg)	615 (578, 652)	603 (570, 636)	504.3 (438.6, 570.0)	461.6 (377.7, 545.5)	611.6 (564.4, 658.8)	593.9 (555.1, 632.8)	772.2, (676.5, 868.0)	748.9 (658.4, 839.4)
Thiamin (mg)	1.5 (1.4, 1.5)	1.3 (1.2, 1.4)	1.4 (1.1, 1.7)	1.1 (1.0, 1.3)	1.4 (1.4, 1.5)	1.3 (1.2, 1.5)	1.8 (1.7, 2.0)	1.4 (1.3, 1.6)
Riboflavin (mg)	1.8 (1.7, 1.8)	1.5 (1.4, 1.5)	1.4 (1.3, 1.5)	1.3 (1.2, 1.4)	1.7 (1.7, 1.8)	1.5 (1.4, 1.5)	2.3 (2.1, 2.5)	1.7 (1.5, 1.8)
Niacin equivalents (mg)	31 (30, 32)	28 (27, 29)	26.5 (24.7, 28.4)	24.0 (21.9, 26.1)	30.7, (29.6, 31.7)	28.1 (27.3, 28.9)	37.8 (35.8, 39.7)	31.3 (29.3, 33.3)
Vitamin B_6 (mg)	2.0 (2.0, 2.1)	1.9 (1.8, 1.9)	1.7 (1.6, 1.8)	1.6 (1.5, 1.8)	2.0 (1.9, 2.1)	1.8 (1.8, 1.9)	2.5 (2.3, 2.7)	2.1 (1.9, 2.2)
Vitamin B_{12} (µg)	4.1 (3.8, 4.3)	3.7 (3.5, 3.9)	3.3 (2.9, 3.7)	3.1 (2.8, 3.4)	3.9 (3.7, 4.2)	3.6 (3.4, 3.8)	5.4 (4.9, 6.0)	4.5 (4.1, 5.0)
Folate (µg)	209 (201, 218)	190 (184, 197)	176.4 (158.5, 194.2)	165.7 (148.1, 183.3)	206.8 (197.6, 216.0)	188.6 (181.1, 196.0)	264.1 (242.4, 285.8)	215.6 (198.1, 233.1)
Vitamin C (mg)	92.0 (83.1, 100.3)	98.0 (90.3, 105.7)	72.5 (56.7, 88.3)	70.5 (50.5, 90.5)	89.3 (79.2, 99.4)	99.9 (90.3, 109.4)	131.0 (99.0, 99.4)	110.2 (90.6, 129.7)
Vitamin D (µg)	2.4 (2.2, 2.6)	2.3 (2.2, 2.4)	2.2 (1.5, 2.8)	2.0 (1.6, 2.4)	2.3 (2.2, 2.5)	2.2 (2.1, 2.4)	3.1 (2.8, 3.5)	3.0 (2.6, 3.3)
Calcium (mg)	891(855, 927)	787 (757, 818)	709.3 (653.2, 765.3)	644.2 (576.6, 711.9)	878.9 (839.0, 918.9)	773.0 (739.1, 807.0)	1152.6 (1049.3, 1256.0)	962.9 (873.2, 1052.6)
Iron (mg)	9.3 (9.0, 9.6)	8.3 (8.1, 8.6)	7.6 (7.0, 8.2)	6.8 (6.1, 7.4)	9.3 (9.0, 9.7)	8.4 (8.1, 8.7)	11.1 (10.4, 11.9)	9.3 (8.7, 10.0)
Zinc (mg)	7.1 (6.9, 7.4)	6.2 (6.0, 6.4)	6.0 (5.5, 6.5)	5.0 (4.6, 5.5)	2.1 (6.8, 7.4)	6.2 (6.0, 6.3)	1.8 (8.3, 9.5)	7.5 (6.9, 8.1)
Selenium (µg)	64 (62, 67)	60 (57, 62)	54.5 (48.7, 60.2)	46.0 (40.0, 52.2)	64.2 (61.0, 67.4)	60.2 (57.8, 62.6)	78.3 (71.3, 85.3)	70.0 (63.2, 76.7)
Iodine (µg)	158 (149, 168)	136 (129, 142)	122.1 (107.3, 136.8)	105.6 (90.2, 121.0)	153.2 (143.0, 163.3)	132.4 (125.5, 139.3)	229.7 (197.3, 265.1)	170.5 (147.3, 193.8)

Values are shown per day (mean with 95% CI).

Under- and over-reporting were identified by the method of Torun [17].

Supplementary Table S4 Comparison of macronutrient intakes between plausible reporters in the IoM and ALSPAC from food records in 7-year-old children by sex

Nutrient	Plausible reporters				P value	
	IoM		ALSPAC		Boys	Girls
	Boys	Girls	Boys	Girls		
n	162	165	2663	2579		
Protein (g)	63.3	58.2	57.4	53.1	≤0.001	≤0.001
Fat (g)	70.0	65.1	70.4	66.0	0.678	≤0.001
Saturated fat (g)	29.2	26.7	28.5	26.5	0.249	0.680
Carbohydrate (g)	256	239	238	219	≤0.001	≤0.001
Energy intake (MJ)	7.76	7.21	7.39	6.84	≤0.001	≤0.001
Free sugars (g)	86.7	83.7	81.6	75.1	≤0.001	≤0.001
Fibre (g NSP)	11.1	10.4	10.8	10.0	0.001	0.060

Supplementary Table S5 Comparison of food group intakes (g/day) between plausible reporters in the IoM and ALSPAC from food records in 7-year-old children, by sex

**Nutrient**	**Plausible reporters**				**P value**	
	**IoM**		**ALSPAC**			
	**Boys**	**Girls**	**Boys**	**Girls**	**Boys**	**Girls**
n	162	165	2663	2579		
**Total bread**	95.6	93.0	74.1	66.9	≤0.001	≤0.001
White bread	78.7	77.4	58.3	53.2	≤0.001	≤0.001
Brown bread	5.4	2.9	2.4	2.4	≤0.001	0.424
Wholemeal bread	7.8	5.2	8.9	7.4	0.550	0.142
**Total milk**	331.9	242.3	279.8	232.1	0.001	0.466
Full-fat milk	207.3	131.3	140.1	111.3	≤0.001	0.110
Semi-skimmed milk	98.2	94.1	131.2	112.0	0.019	0.153
Skimmed milk	13.5	9.8	5.4	5.2	0.003	0.101
**Total meat**	103.4	33.0	81.7	22.8	≤0.001	≤0.001
Pork	7.0	5.4	5.0	4.6	0.096	0.531
Beef	18.6	20.1	13.0	12.5	0.006	≤0.001
Bacon, ham	9.4	8.8	8.2	7.4	0.257	0.143
Lamb	8.7	7.4	5.4	4.8	0.013	0.034
Chicken, turkey dishes	23.0	21.1	16.8	16.3	0.002	0.008
Other potatoes	37.8	36.2	29.5	29.5	0.004	0.015
Chocolate, confectionery	13.7	14.6	16.8	15.6	0.029	0.426
Sugar, confectionery	5.4	6.1	10.7	12.0	0.001	0.001
Fruit juice	119.6	135.2	89.5	89.9	0.007	≤0.001
Soft drinks, sugar-sweetened	131.8	218.8	225.7	111.5	≤0.001	≤0.001
Soft drinks, diet or low calorie	151.8	119.1	338.9	308.4	≤0.001	≤0.001
